# Construction and characterization of a recombinant human adenovirus vector expressing bone morphogenetic protein 2

**DOI:** 10.3892/etm.2013.1162

**Published:** 2013-06-17

**Authors:** ZHENG ZHANG, GUOXIAN WANG, CHEN LI, DANPING LIU

**Affiliations:** 1 Department of Orthopaedics, The First Affiliated Hospital of Liaoning Medical University, Jinzhou, Liaoning 121001;; 2 Department of Pharmacology, Liaoning Medical University, Jinzhou, Liaoning 121000;; 3 Biological Samples Library, The First Affiliated Hospital of Liaoning Medical University, Jinzhou, Liaoning 121001, P.R. China

**Keywords:** bone morphogenetic protein 2, green fluorescent protein, adenovirus vector, construction, alkaline phosphatase

## Abstract

The aim of this study was to construct and characterize a novel recombinant human adenovirus vector expressing bone morphogenetic protein 2 (BMP2) and green fluorescent protein (GFP). The BMP2 gene in the plasmid pcDNA3-BMP2 was sequenced and the restriction enzyme recognition sites were analyzed. Following mutagenesis using polymerase chain reaction (PCR), the gene sequence after the translation termination codon was removed and new restriction sites were added. The mutated BMP2 gene (BMP2^+^ gene) was cloned into an adenovirus shuttle vector to obtain pShuttle cytomegalovirus (CMV)-BMP2^+^-internal ribosome entry site (IRES)-hrGFP-1. The adenovirus plasmid pAd CMV-BMP2^+^-IRES-hrGFP-1 was constructed by homologous recombination and was transfected into HEK293A cells, followed by adenovirus packaging. pAd CMV-BMP2 was used as the control. The two types of adenovirus were transfected into marrow stromal cells (MSCs). The expression of BMP2 and GFP, as well as the alkaline phosphatase (ALP) activity of expressed BMP2 were detected. Following mutagenesis, the BMP2 gene sequence and recombinant adenovirus vector were as predicted. The novel adenovirus vector expressed both BMP2 and GFP, indicating that a novel recombinant human adenovirus vector expressing BMP2 had been successfully constructed.

## Introduction

Bone morphogenetic protein 2 (BMP2) is a cytokine involved in the induction of osteogenic differentiation; it plays a key role in the differentiation of osteogenic progenitor cells to osteoblasts and chondroblasts, as well as in ossification (1–5). However, the direct use of BMP2 has the disadvantages of low biological activity following *in vitro* extraction, susceptibility to dilution and tissue absorption, a short half-life (t_1/2_ <0.1 day) and a complex purification process (6), which has limited its clinical application. Transgenic osteogenesis induction using a BMP2-expressing vector achieves sustained BMP2 expression *in vivo* within a certain period and overcomes the problems associated with the direct use of BMP2; this has attracted widespread attention in recent years. The use of an adenovirus vector is considered the most effective means for conducting transgenic BMP2-induced osteogenesis (7,8). In the present study, in order to facilitate the detection of gene expression, a novel system for the construction of adenoviral vectors was established, in which the BMP2 gene and the green fluorescent protein (GFP) gene were simultaneously expressed. Following mutagenesis, a FLAG epitope tag was attached to the BMP2 gene. The expression levels of the BMP2 and GFP genes in the vectors in marrow stromal cells (MSCs) were detected and the biological activity of expressed BMP2 in the induction of osteogenesis was determined.

## Materials and methods

### Identification of pcDNA3-BMP2 and analysis of restriction enzyme recognition sites

The *E. coli* DH-5α strains containing pcDNA3-BMP2 (provided by Professor Pu Qin, Department of Biochemistry, Fourth Military Medical University, Xi’an, China) were inoculated to LB-ampicillin agar. Then, a single colony was selected. Following amplification, the plasmid was extracted and the *Not*I and *Not*I+*Xba*I restriction enzyme reaction systems were established. The positive clone was identified using agarose gel electrophoresis. The BMP2 gene was sequenced using promoter sequences of T7 and Sp6 RNA polymerase at the two sides of the pcDNA3-BMP2 multiple cloning sites. The restriction enzyme recognition sites in the BMP2 gene were analyzed. Primer synthesis and sequencing were performed by Takara Biomedical Technology, Dalian, China.

### Mutation of the BMP2 gene and subclone of the pcDNA3 plasmid expressing the mutated BMP2 gene (BMP2^+^)

The BMP2 gene was mutated by polymerase chain reaction (PCR), using pcDNA3-BMP2 as the template. The sequence after the translation termination codon TAG (including TAG) in the BMP2 gene was removed and *Xho*I and *Xba*I were added. The PCR conditions were as follows: 30 cycles of 98°C for 5 min, 94°C for 30 sec, 55°C for 30 sec and 68°C for 5 min. The PCR products were obtained. The *Not*I and *Xba*I reaction system was established using pcDNA3-BMP2 and PCR products as substrates, respectively (at 37°C for 2 h). The mutation products were identified by gel electrophoresis. T4 DNA ligase was added to reconnect the mutated BMP2 gene and pcDNA3 (at 16°C overnight). On the following day, the connected reaction solution was transformed into competent *E. coli* DH5α cells (Center Laboratory, First Affiliated Hospital of Liaoning Medical University, Jinzhou, China), followed by inoculation into LB-ampicillin agar. Following amplification, the plasmid was extracted. The positive clone was identified by restriction enzyme reaction and electrophoresis. The pcDNA3-BMP2^+^ plasmid was obtained.

### Construction of the adenovirus shuttle plasmid pShuttle cytomegalovirus (CMV)-BMP2^+^-internal ribosome entry site (IRES)-hrGFP-1

The *Not*I and *Xba*I restriction enzyme reaction system was established using pcDNA3-BMP2^+^ and pShuttle CMV-IRES-hrGFP-1 (Stratagene Corporation, La Jolla, CA, USA) as substrates, respectively (at 37°C for 2 h), followed by identification using agarose gel electrophoresis. The BMP2^+^ gene fragment and pShuttle CMV-IRES-hrGFP-1 fragment were retrieved, respectively, and were connected using T4 DNA ligase (at 16°C overnight). On the following day, the connected reaction solution was transformed into competent *E. coli* DH5α cells, followed by inoculation into LB-ampicillin agar. Following amplification, the plasmid was extracted. The positive clone was identified by restriction enzyme reaction and electrophoresis. The plasmid pShuttle CMV-BMP2 was constructed using the same method as above.

### Construction of the adenovirus plasmid by homologous recombination

Strains containing the plasmid pShuttle CMV-BMP2^+^-IRES-hrGFP-1 and pShuttle CMV-BMP2 were inoculated on LB-kanamycin agar, respectively. The plasmid was extracted and the *Pme*I (New England Biolabs Ltd., Beijing, China) restriction enzyme reaction system was established (at 37°C for 2 h), followed by identification using agarose gel electrophoresis. The adenovirus genome DNA was retrieved by cutting gel and was dissolved in ddH_2_O (final concentration, 50–100 ng/*μ*l) for use. Homologous recombination of the adenovirus plasmid was performed in an electroporation apparatus (200 Ω, 2.5 kV, 25 *μ*F). The recombinant plasmid was extracted. Following the *Pac*I [New England Biolabs (Beijing) Ltd., Beijing, China] restriction enzyme reaction, agarose gel electrophoresis was conducted to identify the positive clone. Finally, the pAd CMV-BMP2^+^-IRES-hrGFP-1 and pAd CMV-BMP2 plasmids were extracted using an Ultrapure Plasmid Purification kit (Novagen Inc., Madison, WI, USA)

### Packaging of the adenovirus

A *Pac*I restriction enzyme reaction system was established. The adenovirus genome DNA was re-dissolved in ddH_2_O (final concentration, 5 *μ*g/225 *μ*l). Conventional resuscitation and subculture of HEK293A cells (American Type Culture Collection, Manassas, VA, USA) were performed until 70% of the cells were fused. The two types of adenovirus genome DNA and transfection reagents were added to cell culture dishes, respectively, followed by 10 h incubation at 37°C, 5% CO_2_. When clear cytopathic phenomenon appeared, the cells were collected and the titers were determined.

### Determination of BMP2 and GFP expression

MSCs (Central Laboratory, First Affiliated Hospital of Liaoning Medical University) were inoculated in a 6-well plate (5×10^5^ cells/well). The cells in two wells were randomly selected for transfection with pAd CMV-BMP2^+^-IRES-hrGFP-1 (experimental group) and the cells in another two wells were transfected with pAd CMV-BMP2 (control group). The multiplicity of infection (MOI) was 50. The remaining two wells were used as blank controls (blank group). GFP was detected under a fluorescence microscope. Total mRNA was extracted using an RNA purification kit (Takara Biomedical Technology) and 80 ng RNA from each group was added to a reaction tube, respectively. Then, 8.5 *μ*l ddH_2_O without RNase was added, followed by denaturation at 75°C for 5 min and cooling on ice. cDNA was synthesized under the following conditions: 30°C for 10 min, 42°C for 30 min and 99°C for 10 min. Following the reaction, cooling on ice was conducted for 5 min. The specific primers for cDNA were designed and synthesized and the reverse transcription (RT)-PCR reaction system was established, with the following reaction conditions: 94°C for 2 min, 94°C for 30 sec, 55°C for 30 sec and 72°C for 1.5 min; 30 cycles. Expression of the BMP2 gene in the three groups was detected.

### Detection of alkaline phosphatase (ALP) activity

MSCs were inoculated in a 96-well plate (1×10^3^ cells/well). The cells in 32 wells were randomly selected for transfection with pAd CMV-BMP2^+^-IRES-hrGFP-1 (experimental group) and the cells in another 32 wells were transfected with pAd CMV-BMP2 (control group). The remaining 32 wells were used as the blank group. Cells in each group (8 wells) were collected on days 6, 8, 10 and 12 after transfection and the cell concentration of each well was adjusted to 1×10^5^ cells/ml. Additionally, 1 ml cell solution in each group was used for detection of ALP activity (kits were purchased from Upstate Biotechnology, Inc., New York, NY, USA).

### Statistical analysis

Statistical analysis was performed using SPSS 11.0 statistical software (SPSS, Inc., Chicago, IL, USA). The Student-Newman-Keuls (SNK)-q test was used for multiple comparisons of ALP activity at different time points after transfection. P<0.05 was considered to indicate a statistically significant difference.

## Results

### Identification of pcDNA3-BMP2 and analysis of restriction enzyme recognition sites

**Figure 1.**
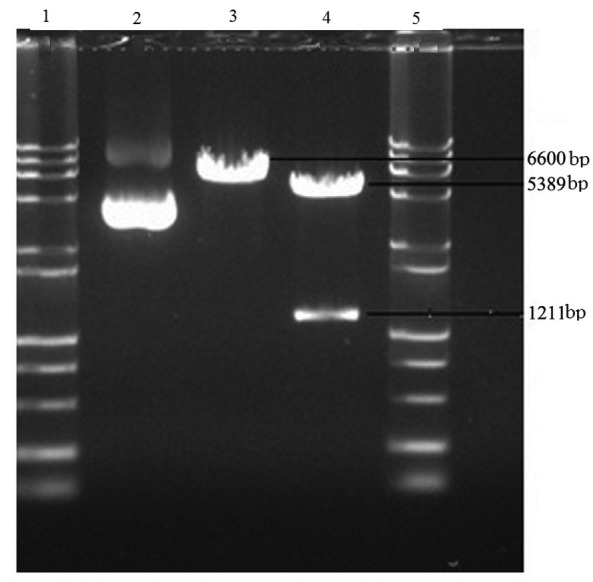
Enzyme electrophoresis of pcDNA3-BMP2. Lanes 1 and 5, DL 15000+2000; lane 2, pcDNA3-BMP2; lane 3, pcDNA3-BMP2/*Not*I; lane 4, pcDNA3-BMP2/*Not*I+*Xba*I. BMP2, bone morphogenetic protein 2.

The results of identification by the *Not*I and *Not*I+*Xba*I restriction enzyme reaction systems demonstrated that the donor plasmid pcDNA3-BMP2 contained the target BMP2 gene (1,211 bp). The sequencing results revealed that the sequence of the BMP2 gene did not contain restriction sites of *Xho*I, *Xba*I, *Pme*I, *Pac*I or *Not*I (Fig. 1). Following gene mutation, the sequences after the translation termination codon were removed and *Xho*I and *Xba*I BMP2 restriction sites were added. The novel plasmid pcDNA3-BMP2^+^ was obtained.

### Construction of the adenovirus shuttle plasmid

**Figure 2.**
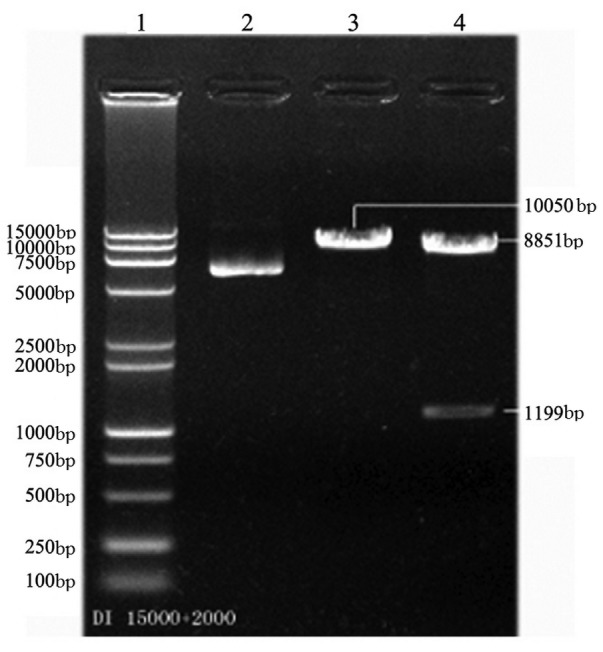
Enzyme electrophoresis of pShuttle CMV-BMP2^+^-IRES-hrGFP-1. Lane 1, DL 15000+2000; lane 2, pShuttle CMV-BMP2^+^-IRES-hrGFP-1; lane 3, pShuttle CMV-BMP2^+^-IRES-hrGFP-1/*Not*I; lane 4, pShuttle CMV-BMP2^+^-IRES-hrGFP-1/*Not*I+*Xba*I. CMV, cytomegalovirus; BMP2, bone morphogenetic protein 2; IRES, internal ribosome entry site; GFP, green fluorescent protein.

Following the *Not*I+*Xba*I restriction enzyme reaction, two bands for pShuttle CMV-BMP2^+^-IRES-hrGFP-1 were obtained by electrophoresis, with molecular weights of 8,851 bp and 1,199 bp, respectively, which were consistent with the theoretical molecular weights of the shuttle vector and BMP2^+^. This indicated the successful construction of pShuttle CMV-BMP2^+^-IRES-hrGFP-1 (Fig. 2).

### Construction of the adenovirus vector

**Figure 3.**
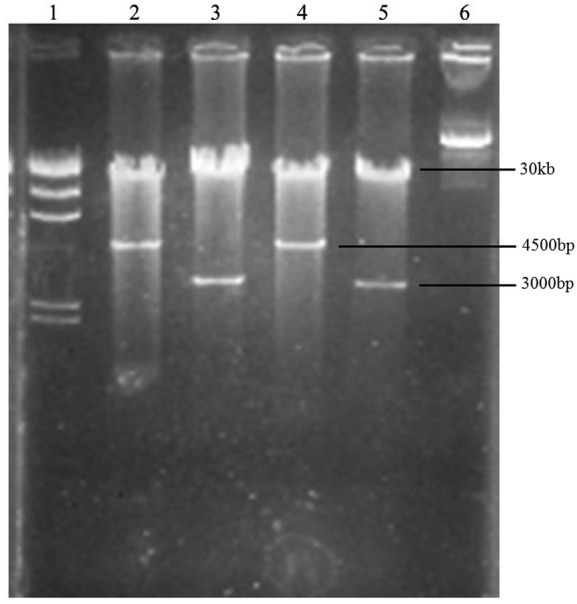
Enzyme electrophoresis of pShuttle CMV-BMP2^+^-IRES-hrGFP-1. Lane 1, λ-*Hind* III digest DNA marker; lane 2-5, pAd CMV-BMP2^+^-IRES-hrGFP-1/*Pac*I (from different clones); lane 6, pAd CMV-BMP2^+^-IRES-hrGFP-1. CMV, cytomegalovirus; BMP2, bone morphogenetic protein 2; IRES, internal ribosome entry site; GFP, green fluorescent protein.

Following the *Pac*I restriction enzyme reaction and agarose gel electrophoresis, two results for the recombinant adenovirus vector were obtained, as follows: i) two bands, at 30 kb and 3,000 bp, respectively, and ii) two bands, at 30 kb and 4,500 bp, respectively. The two results originated from different clones and were in accordance with the theoretical results of the pAd Easy-1 adenovirus system, indicating the successful construction of the adenovirus vector (Fig. 3).

### Packaging of the adenovirus

**Figure 4.**
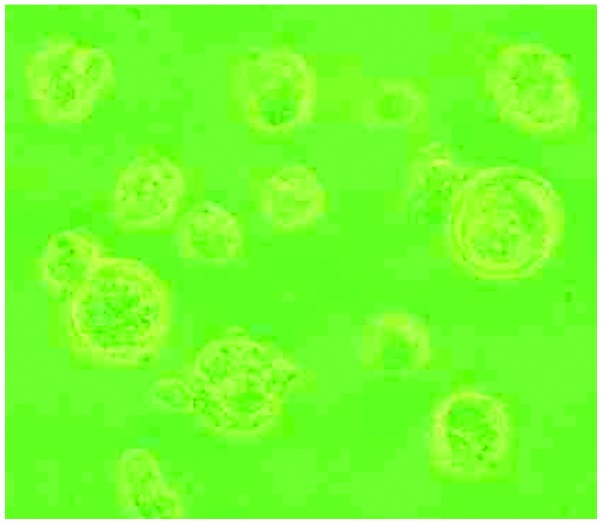
Light microscopy of HEK293A cells packaging the pAd CMV-BMP2^+^-IRES-GFP-1 adenovirus (magnification, ×400). CMV, cytomegalovirus; BMP2, bone morphogenetic protein 2; IRES, internal ribosome entry site; GFP, green fluorescent protein.

The successfully constructed recombinant adenovirus vector was transfected into HEK293A cells for packaging. HEK293A cells presented a cytopathic effect (CPE) with cell suspension, tentacle contraction and swelling, as well as a round shape, indicating successful adenovirus packaging (Fig. 4). Following four rounds of amplification, the titers of the successfully packaged pAd CMV-BMP2^+^-IRES-GFP-1 and pAd CMV-BMP2 were each ∼5×10^8^ pfu/ml.

### Expression of BMP2 and GFP

**Figure 5.**
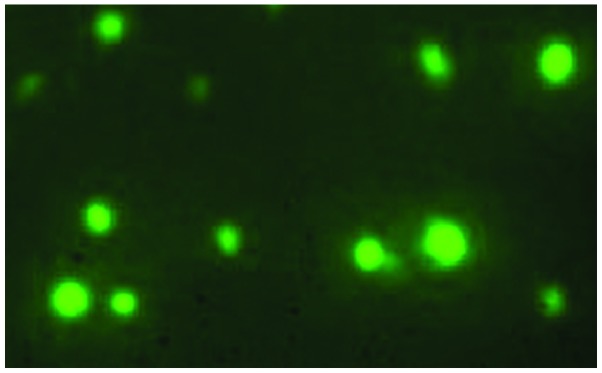
Expression of GFP in adenovirus-transfected MSCs (fluorescence microscopy; magnification, ×400). GFP, green fluorescent protein; MSCs, marrow stromal cells.

**Figure 6.**
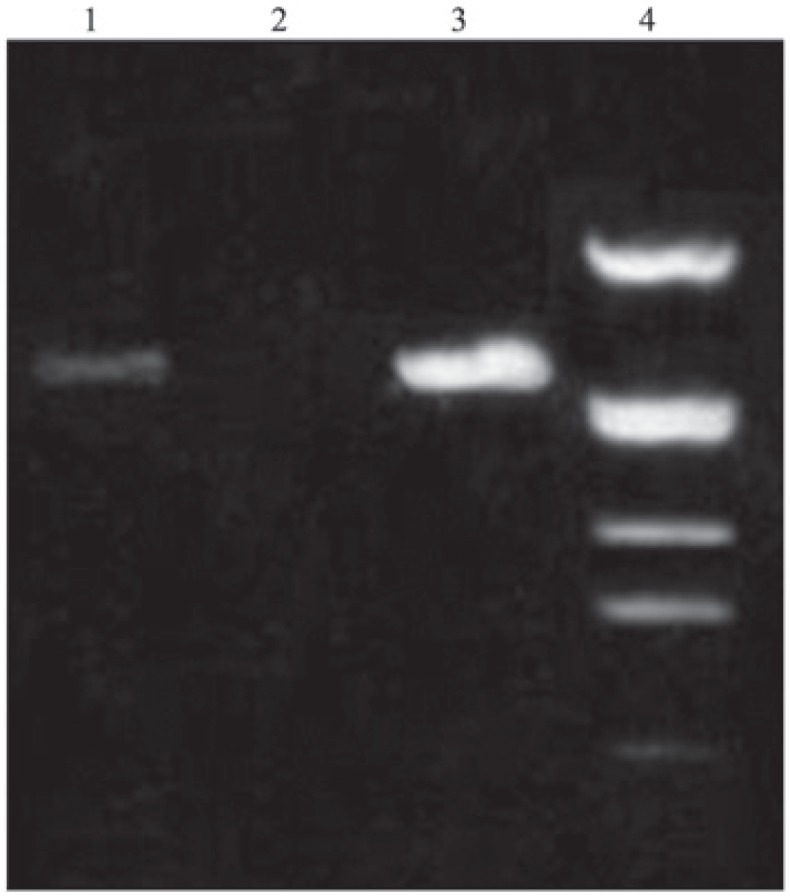
Expression of BMP2 mRNA in adenovirus transfected MSCs (RT-PCR). Lane 1, control group (pAd CMV-BMP2); lane 2, blank group; lane 3, experimental group (pAd CMV-BMP2^+^-IRES-hrGFP-1); lane 4, DL 2000). BMP2, bone morphogenetic protein 2; RT-PCR, reverse transcription-polymerase chain reaction; MSCs, marrow stromal cells; CMV, cytomegalovirus; IRES, internal ribosome entry site; GFP, green fluorescent protein.

From 72 h after transfection of the adenovirus solution into MSCs, fluorescence microscopy revealed high expression levels of GFP, indicating successful transfection (Fig. 5). RT-PCR results demonstrated clear expression of BMP2 mRNA in MSCs in the experimental and control groups, respectively, with no BMP2 mRNA expression in the blank group. This further indicated successful transfection (Fig. 6).

### Detection of ALP activity

Significant differences in ALP activity were observed between the experimental group and the blank group, and between the control group and the blank group (P<0.05; Table I), with no significant difference between the experimental and control groups (P>0.05). This indicated that, compared with pAd-BMP2, pAd-CMV-BMP2^+^-IRES-hrGFP-1 had the same function of inducing osteoblast differentiation; however, this vector included a GFP label, which previous vectors have lacked.

## Discussion

Homologous recombinant adenovirus vectors are an efficient system for expressing the BMP2 gene (9). The constructed vector is essentially a replication-defective recombinant adenovirus from which the E1 gene for DNA replication and viral packaging has been deleted. In the current study, the shuttle vector pShuttle-CMV-IRES-hrGFP-1 was used to construct the novel BMP2-expressing adenovirus vector. There are several important structures, including multiple cloning sites for connecting the target gene, FLAG epitope tag, IRES and GFP behind the strong constitutive CMV promoter. The target gene and the FLAG, IRES and GFP genes are transcribed in polycistronic form at the same time; namely, the four transcripts are located in the same mRNA. With the existence of IRES, the target genes are translated into the target proteins and the ribosome is combined with GFP mRNA, without being dropped from the polycistronic mRNA chain. Therefore, when the target gene is introduced into MSCs, it is simultaneously expressed with GFP (10,11). GFP is easily detected by fluorescence microscopy, immunohistochemistry and other intrusive or non-intrusive detection methods, and it is a useful target gene reporting molecule (12). FLAG is a small synthetic polypeptide that is a type of highly specific antibody, with a translation termination codon in its gene sequence. After removal of the translation termination codon, FLAG may be fused with a target protein and expressed as a type of antigen component of the target protein (13). This has enriched the detection methods of target genes.

In the present study, the restriction enzyme reaction and sequencing were performed on the BMP2 gene of pcDNA3-BMP2, and the restriction enzyme recognition sites were analyzed. Results show that the length of the BMP2 gene is 1,211 bp and the coding sequence is the same as the 318–1,528 base sequence in human BMP2 mRNA (NM 001200) from Genbank; it is mainly composed of the sequence from the translation initiation codon to the translation termination codon (324–1,514 bp). As the BMP2 gene in the donor plasmid has its own translation termination codon, with no restriction site for cloning of the BMP2 gene into pShuttle CMV-IRES-hrGFP-1, PCR is used for mutation of the BMP2 gene. The sequence after the translation termination codon TAG (including TAG) of the BMP2 gene was removed and *Xho*I and *Xba*I were added. The mutated BMP2 gene was subcloned in the pcDNA3 vector and the new donor plasmid pcDNA3-BMP2^+^ was prepared.

Following the successful preparation of the recombinant adenovirus shuttle vector, according to the homologous recombination mechanism in BJ5183 bacteria, the novel adenovirus plasmid pAd CMV-BMP2^+^-IRES-hrGFP-1 was constructed. The *Pac*I restriction map shows that in different recombinant positive clones in BJ5183, the shuttle plasmid and adenovirus plasmid have the DNA prokaryotic replication origin sequence. Following the *Pac*I restriction enzyme reaction, the constructed adenovirus plasmid should produce DNA fragments >23 kb and small fragments at 3.0–4.5 kb. In this study, these two DNA fragments appear in the positive clone of the constructed pAd CMV-BMP2^+^-IRES-hrGFP-1.

In the current study, homologous recombination in bacteria was conducted to construct the adenovirus vector, which saves time and cost. Compared with the widely used homologous recombination method in mammalian cells, this method is simple, fast, economical and efficient. In previous methods (14), homologous recombination has been performed in HEK293A packaging cells and the cell state may greatly affect the recombination efficiency, with a passage number of no more than 45 generations. Occasionally, it has been necessary to use HEK293A cells with a low number of generations. In addition, HEK293A cells are not only the sites for homologous recombination, but also for virus packaging. Following packaging, the virus requires repeated screening, identification and purification. The method in the present study uses bacteria, and the culture, transformation and screening of the recombinant clone are more convenient, economic and faster compared with a method using cells, with more proficient processing. The time from plasmid transformation to appearance of the recombinant clone was only 12–20 h and the total time, including screening time was only 36 h. Most importantly, following identification, the recombinant DNA (adenovirus plasmid) may be directly packaged in HEK293A cells, without secondary screening and identification. In addition, the virus titer and purity of this method are high.

In order to detect the expression of GFP and BMP2, the constructed vector and control vector were transfected into MSCs. After 3 days, the living cells were observed under a fluorescence microscope. Results demonstrated that the MSCs transfected by the constructed vector expressed GFP. Then, the total mRNA of MSCs was extracted for RT-PCR detection. Results of the electrophoresis of PCR products demonstrated that there were DNA fragments with the same length amplified from the two recombinant adenovirus vectors, with no DNA fragment in the blank group. This indicated that the adenovirus vector expressed the BMP2 gene. In order to further confirm the osteogenic induction effect of the expression product (BMP2^+^) and to investigate the kinetics, the ALP activity in MSCs following vector transfection was detected. Results showed that, on the sixth day after transfection, the novel adenovirus vector and control vector significantly increased the ALP activity level in MSCs (P<0.01), with no significant difference between them (P>0.05). Thus, the feasibility of BMP2 expression in a novel adenovirus vector is demonstrated by the biological behavior of expression products.

The BMP2-expressing adenovirus vector constructed in the current study is different from previously prepared BMP2-expressing adenovirus vectors. It not only expresses the target protein BMP2 with the epitope tag, but also expresses the reporting molecule GFP, which provides greater advantages compared with previous vectors. First, introduction of the reporting molecule GFP enables the detection of BMP2 expression in living cells. Therefore, BMP2 expression may be monitored and following detection, the cells may still be used for subsequent experiments. Secondly, the introduction of the FLAG epitope tag enriches the detection means of the target gene, which is particularly important in the absence of specific monoclonal antibodies of the target gene.

Transgenic therapy using cytokines to induce osteogenic differentiation has received widespread attention in the past twenty years. It may cause a revolutionary transformation of bone repair therapy and is likely to bring new hope for bone orthopedic surgery (15–18). The successful construction of a novel BMP2-expressing adenovirus vector is likely to lay a good foundation for further investigation of transgenic therapy for BMP2-induced osteogenic differentiation and bone tissue engineering. This study is a preliminary attempt at the construction of a BMP2-expressing adenovirus vector.

The main aim of this study was to demonstrate the feasibility of the simultaneous expression of GFP and FLAG-labeled BMP2, and to provide a foundation for the investigation of osteogenic differentiation induced by adenovirus-mediated BMP2. However, the newly constructed vector is an adenovirus vector comprising recombinant BMP2 with deletion of the EI and E3 genes. As the vast majority of the viral gene remains in the vector, the immunogenicity and toxicity of the vector remains high. In addition, during the vector packaging process or when the vector enters the human cells with wild virus infection, a virus with replication ability may be generated by homologous recombination.

## Figures and Tables

**Table I.** ALP activity at various time points after transfection (U/l; mean ± SD).

Group	Day 6	Day 8	Day 10	Day 12
Experimental	24.61±0.33	30.86±0.51	33.01±0.64	34.35±0.43
Control	24.50±0.42	29.61±0.53	33.11±0.31	34.01±0.23
Blank	15.68±0.01	16.01±0.54	16.55±0.23	17.67±0.47

Data are presented as mean ± standard deviation. Comparisons among three groups, F=4.32, P<0.05; q_13_=4.67, P<0.01; q_23_=4.64, P<0.01; q_12_=0.47, P>0.05; t=6 days. ALP, alkaline phosphatase.
